# Evaluation of the effect of BioFire FilmArray nested multiplex polymerase chain reaction method on rapid pathogen identification and antimicrobial stewardship in sepsis

**DOI:** 10.1590/1806-9282.20241038

**Published:** 2024-12-16

**Authors:** Tuba Tatlı Kış, Can Biçmen, Suleyman Yıldırım, Ozlem Ediboğlu, Fatma Sebnem Yıldız, Ayriz Tuba Gunduz, Ferhat Demirci, Cenk Kıraklı

**Affiliations:** 1 Health Sciences University, Dr. Suat Seren Chest Diseases and Surgery Training and Research Hospital, Department of Clinical Microbiology and Infectious Diseases – İzmir, Turkey.; 2 Health Sciences University, Dr. Suat Seren Chest Diseases and Surgery Training and Research Hospital, Department of Microbiology – İzmir, Turkey.; 3 Health Sciences University, Izmir Suat Seren Chest Diseases and Surgery Training and Research Hospital, Department of Intensive Care Unit – İzmir, Turkey.; 4 Health Sciences University, Izmir Suat Seren Chest Diseases and Surgery Training and Research Hospital, Department of Medical Biochemistry – İzmir, Turkey.

**Keywords:** Bacteremia, Multiplex PCR, Sepsis

## Abstract

**OBJECTIVE:**

In this study, we aimed to assess the effect of the BioFire FilmArray Blood Culture Identification 2 panel on agent identification and antimicrobial stewardship in patients with a critical state of sepsis secondary to bloodstream infection.

**METHODS:**

This study was designed as a prospective observational study. Patients who developed sepsis and septic shock secondary to bloodstream infection in the intensive care unit were included in the study. Concordance in both monomicrobial and polymicrobial results of Blood Culture Identification 2 panel and conventional blood culture, test result times, and antibiotherapy changes according to Blood Culture Identification 2 panel results were evaluated.

**RESULTS:**

In monomicrobial samples, sensitivity and specificity were 97.1% (95%CI 84.6–99.3) and 100% (95%CI 66.3–100), respectively, for gram-negative pathogens and 85.7% (95%CI 42.1–99.6) and 100% (95%CI 90.2–100), respectively, for gram-positive pathogens. In polymicrobial samples, Blood Culture Identification 2 panel results were 79% in concordance with conventional blood culture results. In this study, when the final turnaround time of the Blood Culture Identification 2 panel was compared with culture results, the Blood Culture Identification 2 panel was on average 1 day, 5 h, and 35 min faster than the culture (p<0.01).

**CONCLUSION:**

Blood Culture Identification 2 testing is a reliable tool for rapid pathogen and antimicrobial susceptibility detection in critically ill sepsis patients. The use of the Blood Culture Identification 2 panel in patients with sepsis and/or septic shock, where the transition to targeted antibiotherapy is critical, may improve patient outcomes.

## INTRODUCTION

Sepsis is one of the most important causes of mortality and morbidity in intensive care units (ICU)^1^. Bloodstream infections (BSI) account for 30–40% of all sepsis cases^2^. Early initiation of antimicrobial treatment is important in the course of sepsis. Inappropriate and inadequate doses of antimicrobial treatment have been reported to increase mortality in sepsis^2,3^. International guidelines recommend the initiation of intravenous antimicrobials within 1 h after the diagnosis of sepsis^4^. Early transition from empirical antimicrobial treatment to targeted antimicrobial treatment requires early detection of the pathogen. However, the most important disadvantage of the conventional blood culture methods used today is their late results. These limitations in the current laboratory methods bring about the need for rapid diagnostic methods in patients with BSI. In recent years, significant progress has been made in the rapid detection and optimal treatment of BSI thanks to the development of multiplex polymerase chain reaction (PCR)-based technologies that can be used to rapidly identify microorganisms grown in blood cultures^5^. Thus, the BioFire FilmArray Blood Culture Identification 2 (BCID2) panel has started to enter our routine practice.

In this study, we aimed to evaluate the effect of the BCID2 panel on agent identification and antimicrobial stewardship in patients with critical sepsis secondary to BSI. There are studies to evaluate the efficacy of the BCID2 panel^6–8^. Our study is important because it evaluates the efficacy of the test and its effect on antimicrobial stewardship in sepsis patients.

## METHODS

### Study design

This prospective observational study was conducted at Dr. Suat Seren Chest Disease and Surgery Training and Research Hospital at the University of Health Sciences between June 2022 and March 2023. Ethics committee approval was obtained from the ethics committee of Dr. Suat Seren Chest Disease and Surgery Training and Research Hospital at the University of Health Sciences (Decision No: 2022/32-44).

### Clinical samples

This study included blood cultures of patients over the age of 18 years who were followed up in the ICU and diagnosed with sepsis and septic shock according to The Third International Consensus Definitions for Sepsis and Septic Shock (Sepsis-3) diagnostic criteria^1^. Of the blood culture bottles taken simultaneously, only the bottle marked as positive was included in the study. Repeated positive blood cultures from the same patient within a 2-week period were excluded from further analysis.

### Microbiological analysis

Blood cultures were analyzed using BacT/Alert automated blood culture device (*bioMérieux, Marcy l’Etoile, France*). Gram staining was performed on all blood culture bottles in which a growth signal was detected and the results were reported. Bacterial identification and drug susceptibility tests were performed at the species level using conventional microbiological methods and an automated Phoenix system (*Becton Dickinson Instrument Systems, Sparks, BD, USA*) by selecting pure colonies grown in passages made on blood, chocolate, and (eosin methylene blue) agar. Antibiogram results were evaluated according to the European Committee on Antimicrobial Susceptibility Testing (EUCAST) recommendations. In addition to microbiological culture and identification procedures, the BCID2 panel [*bioMérieux, Marcy l’Etoile, France*] was used for molecular identification from the blood culture bottle in which a growth signal was detected.

### Reporting times and antimicrobial treatment

For each sample analyzed, the time taken to report the gram stain result of the blood culture bottle to the treating clinicians (hours) and the time taken to report the final results generated by the conventional blood culture method and the BCID2 panel (hours) were evaluated. Adjustment of antimicrobial therapy after the communication of BCID2 panel results was recorded as escalation, de-escalation, or unchanged therapy. De-escalation was defined as narrowing the spectrum of empirically initiated antimicrobial therapy or discontinuation of one of the agents in combination therapy, while escalation was defined as expanding the spectrum of empirically initiated antimicrobial therapy or switching from monotherapy to combined antimicrobial therapy^9^.

### Statistical analysis

Statistical analysis was performed using SPSS for Windows version 26.0. Normality of distribution of continuous variables was checked using the Kolmogorov-Smirnov test. Mean±standard deviation (SD) was given for continuous variables, and number and frequency were given for categorical variables. Student's t-test and Mann-Whitney U test were used to compare continuous variables. Comparison analysis of categorical variables was performed using Pearson's chi-square test and Fisher's exact test. p<0.05 was considered significant. Blood culture was considered the gold standard for monomicrobial and polymicrobial samples, and false positivity, false negativity, specificity, sensitivity, and 95% confidence intervals were calculated using SPSS for Windows version 26.0 accordingly.

## RESULTS

The study included 60 patients with a critical state of sepsis. The mean age of the patients was 68.98 (±12.08) years and 33 (55%) patients were male.

### Distribution of detected agents

Monomicrobial agents were detected in 43 [43/60 (71.6%)] of 60 critically ill sepsis patients with automated blood culture and BCID2 panel. The most common monomicrobial agents were *Klebsiella pneumoniae* [18/43 (41.8%)] and *Acinetobacter baumannii* [5/43 (11.6%)]. In 41 [41/43 (95.3%)] of the monomicrobial samples, the BCID2 panel was consistent with the conventional blood culture method. In two patients [2/43 (4.6%)], discordance between the BCID2 panel and conventional culture methods was detected. This was associated with the detection of microorganisms not included in the BCID2 panel (*Corynebacterium striatum* and *Morganella morgannii*). In monomicrobial samples, the sensitivity and specificity were 97.1% (95%CI 84.6–99.3) and 100% (95%CI 66.3–100), respectively, for gram-negative pathogens and 85.7% (95%CI 42.1–99.6) and 100% (95%CI 90.2–100), respectively, for gram-positive pathogens. The specificity and sensitivity rates calculated according to the agents are shown in Table 1. Polymicrobial agents were detected in 17 patients [17/60 (28.3%)]. BCID2 panel was 79% concordant with conventional blood culture in polymicrobial samples. Detected pathogens and BCID2 panel—conventional blood culture concordance are shown in Table 2.

**Table 1:** Agents detected by Blood Culture Identification 2 panel in monomicrobial samples and specificity–sensitivity rates.

Microorganism	TP (n) [BC+/BCID2+]	FP (n) [BC-/BCID2+]	FN (n) [BC+/BCID2-]	TN (n) [BC-/BCID2-]	SE (%)	95%CI (%)	SP (%)	95%CI (%)
*Enteric bacteria*	21	0	1	21	95.4	77.1–99.88	100	83.9–100
*K. pneumoniae*	18	0	0	25	100	81.5–100	100	86.2–100
*E. coli*	2	0	0	41	100	15.8–100	100	91.4–100
*Proteus* spp.	1	0	0	42	100	2.5–100	100	91.5–100
*E. cloacae complex*	0	0	0	43	NA	–	100	91.8–100
*K. aerogenes*	0	0	0	43	NA	–	100	91.8–100
*K. oxytoca*	0	0	0	43	NA	–	100	91.8–100
*Salmonella* spp.	0	0	0	43	NA	–	100	91.8–100
*S. marcescens*	0	0	0	43	NA	–	100	91.8–100
*A. calcoaceticus-baumannii*	5	0	0	28	100	47.8–100	100	87.6–100
*S. maltophilia*	4	0	0	39	100	39.7–100	100	90.9–100
*P. aeruginosa*	2	0	0	41	100	15.8–100	100	91.4–100
*H. influenzae*	1	0	0	42	100	2.5–100	100	91.5–100
*N. meningitidis*	0	0	0	43	NA	–	100	91.8–100
*Bacteroides fragilis*	0	0	0	43	NA	–	100	91.8–100
Gram negatives, overall	33	0	1	9	97.1	84.6–99.3	100	66.3–100
*Staphylococcus* spp.	1	0	0	42	100	2.5–100	100	91.5–100
*S. epidermidis*	1	0	0	42	100	2.5–100	100	91.5–100
*S. aureus*	0	0	0	43	NA	–	100	91.8–100
*S. lugdunensis*	0	0	0	43	NA	–	100	91.8–100
*Streptococcus* spp.	0	0	0	43	NA	–	100	91.8–100
*S. pyogenes*	0	0	0	43	NA	–	100	91.8–100
*S. pneumoniae*	0	0	0	43	NA	–	100	91.8–100
*S. agalactiae*	0	0	0	43	NA	–	100	91.8–100
*E. faecium*	2	0	0	41	100	15.8–100	100	91.4–100
*E. faecalis*	2	0	0	41	100	15.8–100	100	91.4–100
*L. monocytogenes*	0	0	0	43	NA	–	100	91.8–100
Gram positives, overall	6	0	1	36	85.7	42.1–99.6	100	90.2–100
*C. neoformans/gattii*	1	0	0	42	100	2.5–100	100	91.5–100
*C. albicans*	1	0	0	42	100	2.5–100	100	91.5–100
*C. parapsilosis*	0	0	0	43	NA	–	100	91.8–100
*C. tropicalis*	0	0	0	43	NA	–	100	91.8–100
*C. krusei*	0	0	0	43	NA	–	100	91.8–100
*C. glabrata*	1	0	0	42	100	2.5–100	100	91.5–100
*C. auris*	0	0	0	43	NA	–	100	91.8–100
Yeasts, overall	3	0	0	40	100	29.2–100	100	91.2–100

BCID2: Blood Culture Identification 2; CI: confidence interval; NA: not applicable; TP: true positives; FP: false positives; FN: false negatives; TN: true negatives; BC: blood culture; SE: sensitivity; SP: specificity.

**Table 2:** Pathogens detected in polymicrobial samples and concordance with Blood Culture Identification 2 panel—traditional blood culture.

Polymicrobial samples	BCID2 results	Blood culture results	Concordance
1	*C. tropicalis* *E. faecium*	*C. tropicalis* *E. faecium*	100%
2	*E. faecium* *S. epidermidis* *S. maltophilia*	*E. faecium* *S. epidermidis* *S. maltophilia*	100%
3	*E. faecium* *S. maltophilia*	*E. faecium* *S. maltophilia*	100%
4	*E. faecalis* *A. baumannii*	*E. faecalis* *A. baumannii*	100%
5	*C. glabrata* *S. epidermidis*	*C. glabrata* *S. epidermidis*	100%
6	*C. albicans* *S. epidermidis*	*C. albicans* *S. epidermidis*	100%
7	*A. baumannii* *K. pneumoniae*	*A. baumannii* *K. pneumoniae*	100%
8	*A. baumannii* *E. faecium* *E. faecalis* *S. epidermidis*	*A. baumannii* *E. faecium* *E. faecalis* *S. epidermidis*	100%
9	*E. coli* *C. albicans*	*E. coli* *C. albicans*	100%
10	*E. coli* *K. pneumoniae* *C. glabrata*	*E. coli* *K. pneumoniae* *C. glabrata*	100%
11	*A. baumannii* *S. epidermidis*	*A. baumannii*	50%
12	*K. pneumoniae* *E. faecium* *E. faecalis*	*K. pneumoniae* *E. faecium*	66,6%
13	*E. coli* *K. pneumoniae* *P. aeruginosa* *E. faecalis*	*E. coli* *K. pneumoniae*	50%
14	*A. baumannii* *P. aeruginosa* *E. faecalis*	*A. baumannii* *E. faecalis*	66,6%
15	*E. faecalis* *E. faecium* *S. epidermidis*	*E. faecalis*	3,3%
16	*E. coli* *S. epidermidis*	*E. coli*	50%
17	*E. coli* *S. epidermidis*	*E. coli*	50%
Overall concordance			34/43 (79%)

BCID2: Blood Culture Identification 2.

### Resistance genes detected in Blood Culture Identification 2 panel and phenotypic resistance rates

At least one resistance gene was positive in 40 (31 gram-negative pathogens and 9 gram-positive pathogens) pathogens detected in polymicrobial and monomicrobial samples. Among the *K. pneumoniae* agents (n=22) detected in the BCID2 panel, 20 (90.9%) were positive for at least one resistance gene in the BCID2 panel. Phenotypic resistance was 100% compatible with BCID2 resistance genes for enteric bacteria and gram-positive pathogens. However, while no resistance gene was detected in any of the *A. baumannii* (n=10) pathogens in the BCID2 panel, all pathogens resulted as carbapenem resistant in the conventional blood culture antibiogram. In our study, bla_IMP_, bla_VIM_, and mcr-1 genes were not detected. The concordance between resistance genes detected by the BCID2 panel and phenotypic resistance genes detected by conventional methods is shown in Table 3.

**Table 3:** Concordance between resistance genes detected by Blood Culture Identification 2 panel and phenotypic resistance detected by conventional methods.

Pathogen	Resistance genes detected by BCID2 panel	Resistance Phenotype detected by conventional methods	Concordance (%)
*S. epidermidis*	*MecA/C* (n=6)	Methicillin resistant (n=6)	100%
*E. faecium*	*van A/B*, (n=1)	*Vancomycin resistant*, (n=1)	100%
*E. coli*	bla_CTX-M_, (n=2)	ESBL+, (n=2)	100%
*K. pneumoniae*, (n=20)	bla_ctx-M_+bla_OXA-48_, (n=8)	Carbapenem resistant, (n=8)	100%
	bla_ctx-m_+bla_ndm_+bla_oxa-48_, (n=6)	Carbapenem resistant, (n=6)	100%
	bla_CTX-M_, (n=2)	ESBL+, (n=2)	100%
	bla_KPC_, (n=3)	Carbapenem resistant, (n=3)	100%
	bla_CTX-M_+bla_NDM_ (n=1)	Carbapenem resistant, (n=1)	100%
*Proteus* spp. (n=1)	bla_CTX-M_+bla_OXA-48_, (n=1)	Carbapenem resistant, (n=1)	100%
*A. baumannii* (n=10)	None	Carbapenem resistant, (n=10)	0%
Overall concordance			30/40 (75%)

ESBL: Extended spectrum β-lactamase; BCID2: Blood Culture Identification 2.

### Result times

The mean time taken to communicate the gram stain findings to the clinician was 1925.31 (±966.18) minutes, while the mean time taken to communicate the results generated by the BCID2 panel was 176.38 (±61.8) minutes. Since the BCID2 panel could be run after the gram stain result, it took an average of 2101.70 (±954.24) minutes to transmit the final identification and antimicrobial susceptibility results. With the conventional blood culture method, it took an average of 3876.25 (±1305.41) minutes to deliver the agent identification and antimicrobial susceptibility results. BCID2 panel was 1 day, 5 h, and 35 min faster than the culture (p<0.01). Thus, appropriate antibiotherapy was started earlier, and infection control measures could be taken earlier. According to the BCID2 panel result, 56 (93.3%) of 60 patients had their empirically initiated antibiotherapy changed as a result of joint evaluation with the clinician. Of these, 55 (91.6%) patients had escalation, and 1 (1.6%) patient had de-escalation. In four (6.6%) patients, the empirically initiated antibiotherapy was not changed.

## DISCUSSION

In our study, in monomicrobial samples, the BCID2 panel was 95.3% concordant with the conventional blood culture method. In polymicrobial samples, the concordance of the BCID2 panel with conventional blood culture was lower at 79%. Similar to our study, Berinson et al. found that the BCID2 panel was 88.3% concordant with conventional blood culture in monomicrobial samples, while this rate was 61.3% in polymicrobial samples^10^. In the study by Sparks et al. The BCID2 panel was 92.9% concordant with conventional blood culture in monomicrobial samples, while this rate was 28.6% in polymicrobial samples^11^. In our study, sensitivity and specificity for gram-negative pathogens were 97.1% (95%CI 84.6–99.3) and 100% (95%CI 66.3–100), respectively. The sensitivity and specificity for gram-positive pathogens were 85.7% (95%CI 42.1–99.6) and 100% (95%CI 90.2–100), respectively. In the study conducted by Peri et al., sensitivity and specificity were 100% (95%CI 85.8–100%) and 100% (95%CI 90.3–100%), respectively, for gram-positive bacteria in monomicrobial samples and 100% (95%CI 87.7–100%) and 96.9% (95%CI 83.8–99.9%) for gram-negative bacteria in monomicrobial samples, respectively^12^.

In our study, phenotypic resistance concordant with resistance genes detected in the BCID2 panel was 100% for enteric bacteria and gram-positive bacteria. However, although all pathogens detected for *A. baumannii* were carbapenem resistant, no resistance gene was detected in the BCID2 panel (concordance 0%). Fhooblall et al. reported that the sensitivity rate of *A. baumannii* in the BCID2 panel was below optimal with a rate of 66.7%^13^. In contrast to these findings, Salimnia et al. reported 100% sensitivity for *A. baumannii* in the BCID panel^14^. Rule et al. reported 90% sensitivity for *A. baumannii* in the BCID2 panel^15^. In our study, sensitivity for *A. baumannii* in the BCID2 panel was 100%, but the same success was not achieved in resistance gene detection. *A. baumannii* has many resistance mechanisms such as β-lactamase enzymes, d-aminoglycoside-modifying enzymes, efflux pumps, and porin loss^16^. Since the BCID2 panel can detect bla _IMP_, *bla*_KPC_, *bla*_OXA-48_, *bla*_NDM_, *bla*_VIM_ *, mcr-1*, and *CTX-M* genes for gram-negative bacteria, other *A. baumannii* resistance genes may have been found negative in our study.

Sepsis management requires early detection of the causative pathogen as well as timely administration of appropriate empirical antimicrobial treatment^17^. Rapid and accurate identification of bacteria and fungi causing BSI is essential to optimize antimicrobial choices and ultimately improve clinical outcomes in patients with sepsis and septic shock^4^. In this study, when the final turnaround time of the BCID2 panel was compared with culture results, the BCID2 panel was on average 1 day, 5 h, and 35 min faster than the culture (p<0.01). This suggests that the BCID2 panel has the potential to significantly impact the management of septic patients. In the study by Rule et al. similar to our study, when compared to culture results, the BCID panel gave faster results by a mean of 2 days, 3 h, and 17 min (p<0.001)^18^. In the study by Sparks et al., the final result time was 24.6 (±16.8) hours for the BCID2 panel and 38.2 (±21.9) hours for conventional methods^11^. Similarly, in another study, the median time to reach BCID2 results was 21 h, while the median times for pathogen identification and antibiotic susceptibility testing according to culture-based methods were 42 and 49 h, respectively^19^.

In our study, as a result of joint evaluation with clinicians, the initiated empirical antibiotherapy was changed in 56 (93.3%) of 60 patients according to the BCID2 panel result. Escalation was performed in 55 (98.2%) patients, and de-escalation was performed in 1 (1.7%) of these patients. In the study by Rule et al., 32% of the patients were adjusted according to the BCID panel result, 92% of them were escalated, and 8% were de-escalated^18^. In the study by Sparks et al., it was reported that 45.1% (23/51) of patients underwent treatment modification according to the BCID2 result^11^.

## CONCLUSION

In our study, the BCID2 panel enabled earlier agent identification and detection of resistance genes compared to conventional culture methods. As a result, appropriate antibiotherapy changes and early infection prevention and control measures could be taken earlier than conventional culture methods.
